# Fear of Falling in Older Adults Undergoing Comprehensive Geriatric Care: Results of a Prospective Observational Study

**DOI:** 10.3390/jcm14124366

**Published:** 2025-06-19

**Authors:** Marco Meyer, Andreas Arnold, Thomas Stein, Ulrich Niemöller, Christian Tanislav

**Affiliations:** Department of Geriatrics, Diakonie Hospital Jung Stilling Siegen, Wichernstrasse 40, 57074 Siegen, Germany; andreas.arnold@diakonie-sw.de (A.A.); thomas.stein@diakonie-sw.de (T.S.); ulrich.niemoeller@diakonie-sw.de (U.N.); christian.tanislav@diakonie-sw.de (C.T.)

**Keywords:** older adults, geriatrics, comprehensive geriatric care, fear of falling

## Abstract

**Objectives**: This prospective observational study aimed to investigate the prevalence, progression, and clinical factors associated with fear of falling (FOF) in older adults hospitalized for comprehensive geriatric care (CGC). **Methods**: FOF was assessed using two measures: a single-item question (SIQ) asking, “Are you currently afraid of falling?” with responses scored as (0) not at all; (1) a little; (2) quite a bit; (3) very much, and the Falls Efficacy Scale International (FES-I). FES-I scores were categorized into low (FES-I 16–19), moderate (FES-I 20–27), and high (FES-I 28–64) concerns about falling. FOF scores were analyzed in relation to patients’ characteristics and functional performance. **Results**: A total of 103 patients were included in the final analysis (mean age: 81.9 years, 64.1% female). Upon hospital admission, 74.8% of patients reported FOF (SIQ ≥ 1), with no significant change at discharge (73.8%, *p* > 0.999). Patients’ FES-I scores indicated high concerns about falling, with only slight improvements following CGC. The median FES-I score upon admission decreased from 31 (IQR: 23.5–40) to 30 (IQR: 23.5–38) at discharge (*p* < 0.001). Logistic regression analysis revealed that persistently high concerns about falling (FES-I 28–64) after undergoing CGC were associated with depressive symptoms (Geriatric Depression Scale score ≥ 6; OR: 3.61, 95% CI: 1.30–10.04) and a diagnosis of heart failure (OR: 3.63, 95% CI: 1.30–10.11). Patients’ scores in the Barthel Index, Timed Up and Go Test, and Tinetti Test improved after treatment, but these changes (Δ) did not show a significant correlation with those in the FES-I or SIQ. **Conclusions**: Our findings demonstrate that FOF is highly prevalent among older adults hospitalized for CGC and persists with only minimal improvement following treatment. Persistently high concerns about falling even after completing CGC were associated with depressive symptoms and a diagnosis of heart failure. These results highlight the potential for more targeted interventions within CGC to more effectively address FOF in this vulnerable population.

## 1. Introduction

Due to demographic changes, with an increasing proportion of older adults, falls have become a significant public health concern. A systematic review and meta-analysis of 104 studies, with a total sample size of more than 36 million, revealed that the global prevalence of falls among older individuals is high, at 26.5%, with regional variations observed. Oceania exhibited a fall prevalence of 34.4%, America of 27.9%, Asia of 25.8%, Africa of 25.4%, and Europe reported 23.4% [1]. Numerous risk factors for falls in older adults have been identified, including age, dementia, and balance disorders [2]. Frail older individuals are at a higher risk of falling than those who are prefrail or robust [3,4,5]. Falls among older adults can result in severe, functionally limiting injuries and are often associated with a fear of recurrent falls. Fear of falling (FOF) is a phenomenon with potentially detrimental effects on functional and social aspects of life [6]. This fear may engender a vicious cycle wherein apprehension of falling leads to reduced mobility, further exacerbating the risk of falls and perpetuating the fear [7,8]. Across 153 studies involving over 200,000 participants from 38 countries, the global prevalence of FOF is at a high level, at 49.6%, with a wide range spanning from 6.96% to 90.34%. Among people from developing countries, FOF was more frequent at 53.4%, compared to those from developed countries, where the prevalence was 46.7%. Additionally, it was shown that the prevalence of FOF was higher among patients, at 52.2%, than among community-dwelling individuals, at 48.4% [9]. Furthermore, FOF can be observed in patients both with and without a history of falls, occurring at high levels in 96.7% of those with previous falls and in 75.1% without a history of falls [10]. The prevalence rates can be significantly influenced by the methods used to measure FOF; studies using more comprehensive scales like the Falls Efficacy Scale tend to report higher prevalence compared to those using single-item measures [11]. The etiology of FOF encompasses a multitude of factors, rendering it a significant societal concern and a critical issue in geriatric care settings. A scoping review highlights that older age, female sex, history of previous falls, worse physical performance, and depressive symptoms are associated with FOF [11]. Alcohol consumption also appears to be associated with an increased likelihood of FOF [12]. Given the increasingly aging population, multimorbidity and frailty, in particular, have become a significant concern with potential adverse impacts on the functional capabilities and quality of life of older individuals [13,14]. Both frailty and multimorbidity have been identified as risk factors for falls and FOF, as evidenced by several studies [15,16,17]. A systematic review has also shown that FOF contributes to an increased risk of frailty among community-dwelling older adults [18].

To address common age-related challenges, such as falls, fall-related injuries, frailty, chronic diseases, multimorbidity, and progressive functional limitations, geriatric care approaches have been developed. Patients referred to our department of geriatrics receive comprehensive geriatric care (CGC), a structured, multidisciplinary inpatient treatment program. CGC focuses not only on treating medical conditions but also on addressing rehabilitation needs, aiming to enhance functionality, preserve independence, and improve quality of life. Previous research has demonstrated that patients who received CGC improved in performing basic activities of daily living (ADL) and mobility after treatment [19,20]. Given the patients’ improved functionality, the question arises as to whether FOF may also be reduced following CGC. For these reasons, we aimed to investigate FOF specifically within the context of the holistic treatment provided by CGC. Our focus was on examining the prevalence, progression, and clinical factors associated with FOF among older adults hospitalized for CGC.

## 2. Methods

### 2.1. Study Design, Subjects, and Setting

This monocentric prospective observational study was conducted in the geriatric department of the Diakonie Hospital Jung-Stilling, an acute care and academic teaching hospital located in Siegen, South Westphalia, Germany. Patients were referred to the department of geriatrics through various pathways, including general practitioners, intra-hospital transfers from other departments, emergency admissions, and referrals from other hospitals in the surrounding region. The geriatric department provides care for a broad range of older patients, including those with acute medical conditions, individuals recovering from surgery, and patients presenting with prevalent geriatric syndromes [21].

After obtaining informed consent, patients were enrolled in the study. Inclusion criteria for study participation comprised hospitalization in the geriatric department and provision of written informed consent. Exclusion criteria included treatment outside the geriatric department, absence of signed informed consent, and moderate to severe cognitive impairment, defined as a Mini Mental State Examination score below 24 points.

The study received approval from the local ethics committee and was prospectively registered in the German Clinical Trials Register.

### 2.2. Comprehensive Geriatric Care

Comprehensive Geriatric Care (CGC) is implemented in our department of geriatrics as a structured, multidisciplinary inpatient treatment program in accordance with the criteria defined by OPS code 8-550 of the German Operation and Procedure Classification System. This code specifies detailed structural, procedural, and documentation requirements that the performing clinic must meet for the procedure to be reimbursable by health insurance. An essential aspect of CGC is the comprehensive geriatric assessment (CGA). Basic ADLs are assessed using the Barthel Index (BI), walking ability is measured with the Timed Up and Go Test (TUG), and balance and gait are evaluated through the Tinetti Balance and Gait Test (TBGT) [22,23,24,25]. BI, TUG, and TBGT are assessed both upon hospital admission and again at discharge as measures of functional outcomes following receipt of CGC. Upon admission, cognitive and emotional evaluations are conducted using the Mini Mental State Examination (MMSE), Shulman’s Clock-Drawing Test (CDT), and Geriatric Depression Scale (GDS, 15-item questionnaire) [26,27,28,29,30]. In addition, a structured social assessment is performed to evaluate aspects such as the patient’s social environment, housing, and living conditions, and the need for assistive devices and care services. CGC hospitalization in our department is planned to last at least two weeks and includes 20 treatment sessions, each lasting 30 min. The treatment sessions cover a broad spectrum of therapies, including physiotherapy, occupational therapy, speech therapy, and psychological support. After a thorough assessment of the patient’s medical condition and functional limitations, a multidisciplinary team—consisting of experienced geriatricians, nursing staff, psychologists, social workers, and therapists specializing in occupational, physical, and speech therapy—collaborates to create a tailored treatment plan for each individual. The patient’s progress is regularly evaluated in weekly multidisciplinary team meetings, where the treatment strategy is reviewed and adjusted to optimally address the patient’s specific deficits.

### 2.3. Outcome Measures After Comprehensive Geriatric Care

#### 2.3.1. Barthel Index

The Barthel Index (BI) is a standardized instrument used to assess a patient’s level of functional independence in ten basic ADL. These ADLs are categorized into self-care and mobility tasks, such as bathing, toileting, dressing, eating, mobility, as well as bowel and bladder control. The BI assigns a score ranging from 0 to 100, with higher scores indicating a higher level of independence and greater autonomy in executing these activities independently [22,31].

#### 2.3.2. Tinetti Balance and Gait Test

The Tinetti Balance and Gait Test (TBGT) is a clinical tool used to assess both balance and gait performance. It comprises a balance component, which includes tasks such as standing up from and sitting down on a chair, and a gait component, which evaluates walking patterns, step symmetry, and trunk stability. The maximum score achievable is 28 points, with higher scores indicating better functional performance [24,25].

#### 2.3.3. Timed Up and Go Test (TUG)

The Timed Up and Go Test (TUG) is a clinical measure used to assess an individual’s walking ability and fall risk. In this test, the patient starts seated in a chair, stands up, walks three meters, turns around, walks back to the chair, and sits down. The total time taken to complete these actions is recorded, with longer durations reflecting reduced mobility and a higher risk of falling [23]. We categorized the TUG test results as follows: Category 5 represents patients unable to walk; Category 4 includes those taking more than 30 s to perform the TUG; Category 3 comprises individuals completing the TUG in 20–29 s; Category 2 involves completion of the TUG in 10–19 s; and Category 1 indicates completion of the TUG in less than 10 s.

#### 2.3.4. Assessment of Fear of Falling (FOF)

Patients’ concerns about falling were assessed with a single-item question (“Are you currently afraid of falling?”) and Falls Efficacy Scale International (FES-I) upon hospital admission and at discharge. Score from single-item question was documented on a 4-point scale: (0) not at all; (1) a little; (2) quite a bit; and (3) very much, using response categories previously described for assessing FOF [32].

The FES-I is a questionnaire comprising 16 items that specifically address a range of social and physical activities in daily life. Individuals taking the FES-I are prompted to indicate their level of concern regarding the possibility of falling while engaging in these activities. The scores on the FES-I questionnaire span from a minimum of 16 points to a maximum of 64 points, with higher scores indicating severe concerns about falling. The FES-I employs a four-point scale, where each point corresponds to a score, with 1 = “not at all concerned”; 2 = “somewhat concerned”; 3 = “fairly concerned”; and 4 = “very concerned” [33,34]. Cut-off points have been defined for FES-I, categorizing scores as follows: 16–19 indicating low concerns, 20–27 indicating moderate concerns, and 28–64 indicating high concerns [35].

### 2.4. Statistical Analyses

Data are presented as mean and standard deviation, median and interquartile range, as well as counts and percentages. Normal distribution was assessed using the Shapiro–Wilk test. To compare two dependent samples, the Wilcoxon signed-rank test was used. Categorical variables were analyzed using the McNemar test for dependent categorical variables. Spearman’s rank correlation coefficient (Spearman’s rho) was calculated to assess the strength and direction of associations between variables in the correlation analysis. Changes in assessment scores (Δ) were calculated as the difference between discharge and admission scores. Logistic regression was performed to identify clinical factors associated with persistently high concerns about falling after completing CGC, defined as patients scoring between 28 and 64 on the FES-I at both admission and discharge. The logistic regression model includes sex, heart failure diagnosis, TBGT, and GDS as independent variables. A Tinetti score ≤ 18 (indicating a high risk of falling) [25,36,37] and a GDS score ≥ 6 (indicating depression) [37,38] were selected as cut-off values for dichotomization. A *p*-value < 0.05 was considered significant. Statistical analyses were conducted using Microsoft Excel 2016 (Microsoft Corporation, Redmond, WA, USA), statistical software PSPP (version 1.4.1, GNU project), and jamovi (The jamovi project (2022). jamovi (version 2.3) [computer software]. Retrieved from https://www.jamovi.org).

For the sample size calculation, a Wilcoxon signed-rank test for paired samples was used. Considering an estimated small to moderate change in FOF after undergoing CGC, Cohen’s dz was set at 0.3, with a power of 0.8, and an alpha level of 0.05, resulting in a sample size of 94. To account for an assumed dropout rate of 20%, the planned sample size was set at 113. Sample size calculation was performed using G*Power (version 3.1.9.6) [39,40].

## 3. Results

In this prospective observational study, a total of 111 older adults were initially enrolled. Data from eight participants were excluded due to an MMSE score of <24 (n = 1), incomplete data (n = 1), treatment discontinuation at the patient’s request (n = 3), and treatment discontinuation for medical reasons (n = 3). Consequently, the final sample comprised 103 patients (mean age: 81.9 ± 5.6 years, of whom 64.1% were female). The median hospital stay was 16 (IQR: 16–18) days, and patients received a median of 21 (IQR: 20–26) treatment units. Comorbidities are summarized in Table 1. Upon hospital admission, patients’ functional status was assessed using the GDS (median: 3, IQR: 2–6), MMSE (median: 28, IQR: 27–29), and CDT (median: 2, IQR: 2–3). The BI was median 55 (IQR: 45–65) upon admission and increased to 80 (IQR: 62.5–85) at discharge (*p* < 0.001). The TUG improved from median 4 (IQR: 3–4) to 3 (IQR: 2–4) (*p* < 0.001), and TBGT from median 14 (IQR: 8–18) to 20 (IQR: 14–23) (*p* < 0.001).

FOF was evaluated using the SIQ and FES-I. Upon admission, 74.8% of patients reported FOF (SIQ ≥ 1), which remained largely unchanged at discharge (73.8%, *p* > 0.999). The median SIQ score upon admission was 1 (IQR: 0.5–2) and remained unchanged at discharge (median 1 (IQR: 0–2), *p* = 0.188). The FES-I score improved after CGC from a median of 31 (IQR: 23.5–40) to a median of 30 (IQR: 23.5–38) (*p* < 0.001) (Table 2).

**Figure 1:**
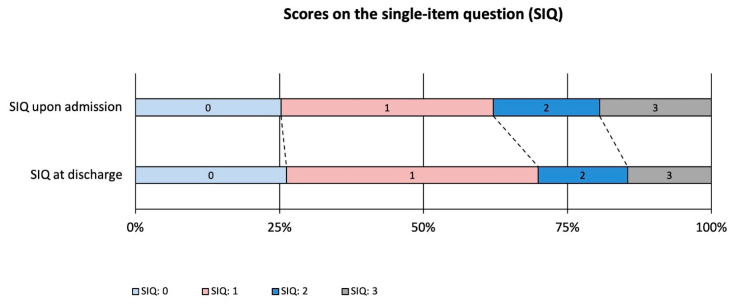
Course of fear of falling among older adults receiving comprehensive geriatric care (CGC). Single-item question, SIQ: “Are you currently afraid of falling?” SIQ 0: not at all, SIQ 1: a little, SIQ 2: quite a bit, and SIQ 3: very much.

**Figure 2:**
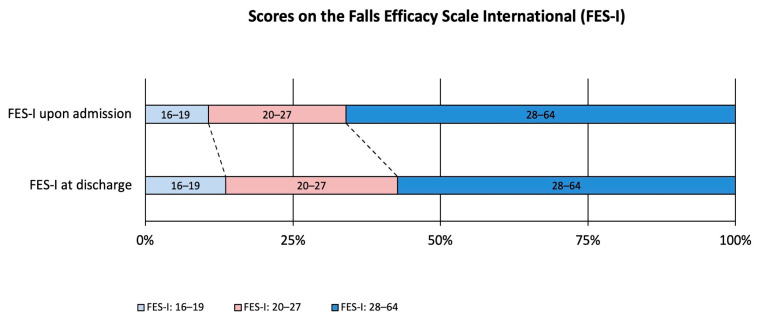
Course of fear of falling among older adults receiving comprehensive geriatric care (CGC). Falls Efficacy Scale International (FES-I) is categorized into low concerns (FES-I: 16–19), moderate (FES-I: 20–27), and high concerns about falling (FES-I: 28–64).

The distribution of SIQ scores at admission was as follows: 25.2% scored 0, 36.9% scored 1, 18.4% scored 2, and 19.4% scored 3. By discharge, 26.2% scored 0, 43.7% scored 1, 15.5% scored 2, and 14.6% scored 3 (Figure 1). The proportion of patients with low concerns about falling (FES-I 16–19) increased from 10.7% at admission to 12.6% at discharge. Moderate concerns (FES-I 20–27) rose from 23.3% to 27.2%, while high concerns (FES-I 28–64) decreased from 66.0% to 60.2% (Figure 2).

Correlation analysis revealed a significant positive correlation between changes in FES-I (Δ FES-I) and SIQ (Δ SIQ) after undergoing CGC (Spearman’s rho = 0.535, *p* < 0.001). However, no significant correlations were found between Δ FES-I and ∆ BI (Spearman’s rho = 0.091, *p* = 0.360), Δ TBGT (Spearman’s rho = −0.073, *p* = 0.463), and Δ TUG (Spearman’s rho = −0.014, *p* = 0.886). Similarly, Δ SIQ did not correlate significantly with Δ BI (Spearman’s rho = 0.106, *p* = 0.286), Δ TBGT (Spearman’s rho = −0.081, *p* = 0.418), and Δ TUG (Spearman’s rho = −0.042, *p* = 0.672) (Table 3).

The logistic regression model includes sex, heart failure diagnosis, TBGT, and GDS as independent variables for predicting persistently high concerns about falling. The model was statistically significant, χ^2^(4) = 19.8, *p* < 0.001, and revealed that a heart failure diagnosis (OR: 3.63, 95% CI: 1.30–10.11) and depressive symptoms (GDS ≥ 6) (OR: 3.61, 95% CI: 1.30–10.04) were associated with persistently high concerns about falling after undergoing CGC (Table 4).

## 4. Discussion

In the presented prospective study, 74.8% of the patients reported a fear of falling (FOF) upon admission for comprehensive geriatric care (CGC). The level of FOF, as assessed in the Falls Efficacy Scale International (FES-I), was high at the beginning of treatment and remained high even at discharge.

Comparing the high frequency of FOF in our study to previous investigations, a recent systematic review and meta-analysis by Xiong et al., which examined the global prevalence of FOF across 153 studies from 38 countries, revealed a worldwide FOF prevalence of 49.6%. This review noted substantial variability in FOF frequency across different populations. Beyond geographic differences, a higher proportion of FOF was observed in patients compared to community-dwelling persons [9]. In a study similar to ours, focusing on older adults undergoing rehabilitation (median age of 82.4 years with various admission diagnoses), a 62.5% prevalence of FOF was reported upon admission for treatment, which increased to 82.1% after discharge [41]. In our study, the FOF levels remained relatively unchanged after undergoing CGC. Median SIQ score did not differ, and only a minimal change in FES-I score at discharge from CGC was observed. This point raises important considerations regarding the impact of CGC interventions on addressing FOF. While CGC encompasses various components aimed at improving the functional status and quality of life of older patients, our results suggest that the therapeutic program of CGC is not specifically tailored for the treatment of FOF. One possible explanation for the limited improvement of FOF after CGC could be the complexity of FOF as a multifaceted phenomenon influenced by various physical, psychological, social, and environmental factors [11,42,43]. Although CGC addresses several of these key determinants, notably the improvement of physical function and mobility, the psychological components contributing to FOF, such as dysfunctional beliefs, over-pessimistic thinking, or low perceived self-efficacy, may receive less attention or lack specific targeting [44,45]. As demonstrated by our study, depressive symptoms were significantly associated with FOF, even after comprehensive treatment like CGC. Previous research has substantiated that psychologic interventions, particularly cognitive behavioral therapy, either alone or in combination with exercise therapy, are beneficial in treating FOF and may reduce it [45,46]. A systematic review and meta-analysis conducted by Liu et al. suggests that cognitive behavioral therapy, with its core components of cognitive restructuring, personal goal setting, and promotion of physical activities, has beneficial effects on FOF both immediately and with retention up to 12 months post-intervention. Individual approaches seem to be more effective compared to group-based cognitive behavioral interventions for reducing FOF [45].

In addition to cognitive behavioral therapy, the impact of exercise therapies such as yoga, Tai Chi, balance training, and strength and resistance training on FOF has also been explored. The results of a previous systematic review and meta-analysis by Kumar et al. indicate an association of performing those treatments with a small to moderate reduction in FOF immediately after the intervention [47]. Supplementary to the results of Kumar et al., regarding the small to moderate effect size of exercise interventions on reduction in FOF, our study reveals that, even after completing an extensive inpatient treatment program like CGC, just marginal improvements in FOF were observed. An adaptation of the rehabilitation measures of CGC could counteract these issues, as demonstrated by a randomized controlled trial investigating the effects of augmenting geriatric inpatient rehabilitation with an additional six-component intervention (“Step by Step” protocol) aimed at addressing falls-related self-efficacy and physical activity [48]. It consists of eight individual sessions during rehabilitation, four telephone calls, and a home visit over a period of 2 months after discharge from inpatient treatment [49]. The study conducted by Pfeiffer et al. revealed that patients who received geriatric inpatient rehabilitation along with additional interventions from their “Step by Step” program exhibited improved falls-related self-efficacy compared to the control group. Moreover, adapting geriatric rehabilitation with measures from “Step by Step” had favorable effects on patients’ physical performance, the number of falls, and their perceived ability to manage falls [48].

Irrespective of the efficacy of the aforementioned treatment options, FOF must first be recognized as a relevant issue for the individual patient. The integration of a standardized assessment of FOF into the comprehensive geriatric assessment could be considered as the first and one of the decisive steps in addressing FOF and serves as the basis before initiating more targeted psychological and exercise interventions within the CGC framework. A specific assessment of FOF not only allows for a more nuanced understanding of individual patients’ needs regarding FOF but also facilitates tailored treatment strategies aimed at mitigating FOF and improving overall outcomes following CGC. Incorporating FOF evaluations into comprehensive geriatric assessment may also help to identify individuals at risk and initiate appropriate interventions sooner, potentially preventing an escalation of FOF and its associated complications. In our study, we observed a discrepancy in the level of FOF across the assessments conducted. Specifically, the median score on the SIQ indicates some FOF, whereas patients’ median score on the FES-I indicates high concerns about falling on admission and at discharge for CGC. In addition to our results, a review by MacKay et al. demonstrated disparities in FOF prevalence across the measures used for assessing FOF, with a higher prevalence in studies using more comprehensive assessments. MacKay and co-workers revealed a mean prevalence of FOF in investigations using a single-item question with 37.38% compared to those that used the Falls Efficacy Scale with 52.31% [11]. Overall, potential discrepancies in the level and prevalence of FOF across the different assessments should be considered when evaluating FOF in clinical care.

A specific assessment of FOF also represents a valuable measure for predicting rehabilitation outcomes. It has been shown that falls-related self-efficacy, assessed via FES-I score, is an independent predictor of functional outcomes at discharge from inpatient geriatric rehabilitation and a four-month follow-up [50]. An inverse association between scores on the FES-I and patients’ performance in coping with basic and instrumental ADL, as assessed by the Barthel Index and Frenchay Activity Index, has been demonstrated in longitudinal follow-up examinations after the completion of rehabilitation measures [41,50].

FOF not only serves as a predictor of functional outcomes following rehabilitation, but, conversely, functional scores, as routinely obtained during CGC, can be predictive of the development of FOF. A cross-sectional study considering older women with type 2 diabetes mellitus has shown that the likelihood of experiencing FOF increased by 1.34 times for a one-point increase in the Geriatric Depression Scale and by 1.36 times for every second longer completing the Timed Up and Go Test [51].

In summary, our study offers an overview of the prevalence and clinical associations of FOF among older adults receiving a comprehensive geriatric inpatient treatment.

A strength of the presented investigation is the presentation of real-world data from everyday clinical practice involving a diverse patient population treated in a hospital geriatrics department. Given the increasing prevalence of FOF with age, the study focuses on a population particularly susceptible to FOF, with an average age of 81.9 years. As a limitation of the study, a selection bias could be considered. Inclusion was based on patients’ readiness to participate, which was assessed upon admission for treatment, and only those who provided consent were enrolled. Individuals with a greater sensitivity to the subject or those more affected by FOF might have been more inclined to participate. Furthermore, the generalizability of our findings is limited, as the results are based on a single-center analysis and exclude patients with moderate to severe cognitive impairment. Additionally, the assessment of FOF was restricted to a hospitalization period with a median duration of 16 days. Nevertheless, the limitations of our study provide important directions for future research. Multi-center studies could be conducted to evaluate the generalizability of our findings across broader populations, and extended follow-up periods could offer valuable insights into the longitudinal development of FOF in patients who have received CGC.

## 5. Conclusions

Upon admission for CGC, three-quarters of the patients reported experiencing FOF. A detailed assessment of FOF related to social and physical activities, as measured by the FES-I, revealed high concerns about falling, with only slight improvement after treatment. This highlights the potential for more targeted interventions within CGC to more effectively address FOF in this vulnerable population.

## Figures and Tables

**Table 1:** Patients’ characteristics.

	(n = 103)
**Age** (mean ± SD, years)	81.9 ± 5.6
**Sex**	
female	66 (64.1%)
male	37 (35.9%)
**Comorbidities**	
Hypertension	88 (85.4%)
Current fracture	50 (48.5%)
Coronary heart disease	36 (35.0%)
Atrial fibrillation	35 (34.0%)
Diabetes mellitus	33 (32.0%)
Heart failure	30 (29.1%)
Carcinoma/Tumor/Leukemia/Lymphoma	25 (24.3%)
Osteoporosis	17 (16.5%)
Chronic obstructive pulmonary disease	16 (15.5%)
Structural brain lesion ^‡^	14 (13.6%)
Peripheral arterial disease	12 (11.7%)
Rheumatic diseases and conditions	6 (5.8%)
Parkinson’s disease	4 (3.9%)
Asthma	3 (2.9%)
**Functional assessments**	
Barthel Index upon admission ^†^	55 (45–65)
Barthel Index at discharge ^†^	80 (62.5–85)
Timed Up and Go Test upon admission ^†^	4 (3–4)
Timed Up and Go Test at discharge ^†^	3 (2–4)
Tinetti Balance and Gait Test upon admission ^†^	14 (8–18)
Tinetti Balance and Gait Test at discharge ^†^	20 (14–23)
Geriatric Depression Scale ^†^	3 (2–6)
Mini Mental State Examination ^†^	28 (27–29)
Shulman’s Clock-Drawing Test ^†^	2 (2–3)
**Hospital stay**	
Hospitalization (days) ^†^	16 (16–18)
Treatment units ^†^	21 (20–26)

^†^ presented as median and interquartile range (IQR) ^‡^ includes acute and previous stroke/intracranial hemorrhage, intracranial tumor, and unspecified head injuries.

**Table 2:** Outcome measures in older adults after undergoing comprehensive geriatric care (CGC).

Assessment	Admission	Discharge	*p*-Value
Falls Efficacy Scale International ^†^	31 (23.5–40)	30 (23.5–38)	<0.001
Single-item question ^†^	1 (0.5–2)	1 (0–2)	0.188
Barthel Index ^†^	55 (45–65)	80 (62.5–85)	<0.001
Timed Up and Go Test ^†^	4 (3–4)	3 (2–4)	<0.001
Tinetti Balance and Gait Test ^†^	14 (8–18)	20 (14–23)	<0.001

^†^ presented as median and interquartile range (IQR).

**Table 3:** Correlation analysis of changes in Falls Efficacy Scale International (Δ FES-I), single-item question (Δ SIQ), Barthel Index (Δ BI), Tinetti Balance and Gait Test (Δ TBGT), and Timed Up and Go Test (Δ TUG) after undergoing comprehensive geriatric care (CGC).

		Δ FES-I	Δ SIQ	Δ BI	Δ TBGT	Δ TUG
**Δ FES-I**	Spearman’s rho					
	*p*-value					
**Δ SIQ**	Spearman’s rho	0.535 ***				
	*p*-value	<0.001				
**Δ BI**	Spearman’s rho	0.091	0.106			
	*p*-value	0.360	0.286			
**Δ TBGT**	Spearman’s rho	−0.073	−0.081	0.298 **		
	*p*-value	0.463	0.418	0.002		
**Δ TUG**	Spearman’s rho	−0.014	−0.042	−0.376 ***	−0.543 ***	
	*p*-value	0.886	0.672	<0.001	<0.001	

* *p* < 0.05, ** *p* < 0.01, *** *p* < 0.001.

**Table 4:** Logistic regression analysis: factors associated with persistently high concerns about falling after undergoing comprehensive geriatric care (CGC) (patients who scored FES-I 28–64 upon admission and at discharge).

Variables	Estimate	Standard Error	*Z*	*p*-Value	OR	95% CI	
						Lower	Upper
Sex (Female)	0.70	0.46	1.51	0.130	2.01	0.81	4.94
Heart failure	1.29	0.52	2.46	0.014	3.63	1.30	10.11
TBGT score ≤ 18 prior to CGC	0.86	0.55	1.59	0.113	2.37	0.82	6.91
Depressive symptoms (GDS ≥ 6)	1.29	0.52	2.47	0.014	3.61	1.30	10.04

OR: odds ratio; 95% CI: 95% confidence interval, TBGT: Tinetti Balance and Gait Test, GDS: Geriatric Depression Scale, CGC: comprehensive geriatric care.

## Data Availability

Data are not publicly available due to privacy and ethical restrictions. Reasonable requests may be directed to the corresponding author.
